# Cerebral Blood Flow Dynamics and Head-of-Bed Changes in the Setting of Subarachnoid Hemorrhage

**DOI:** 10.1155/2013/640638

**Published:** 2013-11-25

**Authors:** David K. Kung, Nohra Chalouhi, Pascal M. Jabbour, Robert M. Starke, Aaron S. Dumont, H. Richard Winn, Matthew A. Howard, David M. Hasan

**Affiliations:** ^1^Department of Neurosurgery, University of Iowa Hospitals and Clinics, 200 Hawkins Drive, JCP 1616, Iowa City, IA 52242, USA; ^2^Department of Neurosurgery, Thomas Jefferson University and Jefferson Hospital for Neuroscience, Philadelphia, PA, USA; ^3^Department of Neurological Surgery, University of Virginia School of Medicine, Charlottesville, VA, USA

## Abstract

Head-of-bed (HOB) elevation is usually restricted in patients with aneurysmal subarachnoid hemorrhage (SAH). The goal of this study is to correlate HOB changes (0° and 90°) with cerebral blood flow using transcranial Doppler (TCD) and thermal diffusion probe in SAH patients. Thirteen patients with SAH were prospectively enrolled in the study. Eight patients underwent placement of a thermal diffusion probe for regional CBF measurement. CBF values were measured with the patients in flat (0°) and upright sitting positions (90°) at days 3, 7, and 10. The average increase in blood flow velocity when changing HOB from 0° to 90° was 7.8% on day 3, 0.1% on day 7, and 13.1% on day 10. The middle cerebral artery had the least changes in velocity. The average regional CBF measurement was 22.7 ± 0.3 mL/100 g/min in the supine position and 23.6 ± 9.1 mg/100 g/min in the sitting position. The changes were not statistically significant. None of the patients developed clinical cerebral vasospasm. Changing HOB position in the setting of SAH did not significantly affect cerebral or regional blood flow. These data suggest that early mobilization should be considered given the detrimental effects of prolonged bed rest.

## 1. Introduction

Patients who suffer aneurysmal SAH are at risk of secondary injuries including cerebral edema and delayed cerebral vasospasm. Traditionally, as a part of the overall treatment protocol for SAH, patients are kept in prolonged bed rest. The assumption is that bed rest will help maintain adequate blood flow to the brain. However, the data supporting this assumption are limited [1].

Blood flow to the brain is critical and complex. CBF is influenced by multiple factors including systemic arterial pressure, distance of the head above the heart, venous and CSF drainage, and vascular tone of cerebral vessels [2]. In a normal individual, as the head is raised, the systemic arterial pressure is maintained by blood pressure reflexes. At the same time, the arterial perfusion pressure to the head is reduced by the distance the head is raised above the heart, but the intracranial pressure is also reduced because of the improved venous drainage. Together with an intact autoregulation response of the cerebral vasculature, the net effect is little change in CBF [3–5]. However, in patients with impaired autoregulation or with vasospasm following SAH, a raise in head position may theoretically diminish CBF. Conversely, in the case of significant cerebral edema after SAH, it may be important to raise the head to improve venous drainage and maximize cerebral perfusion pressure.

Prolonged bed rest, particularly in the elderly and the critically ill, carries its own morbidity [6]. Extensive research has documented the deleterious effects of prolonged bed rest in multiple organ systems, including cardiovascular, musculoskeletal, cognitive, hematologic, and respiratory [7–10]. Significant physiological deterioration begins on the first few days of bed rest. These complications add to the already devastating neurologic injury incurred by SAH.

Considering the potential deleterious effects of prolonged bed rest and its dubious benefit in maintaining cerebral blood flow, we investigated the effect of head position on cerebral blood flow in SAH patients. We hypothesize that the routine practice of placing SAH patients in prolonged bed rest is unnecessary to maintain stable CBF.

## 2. Material and Methods

**Figure 1:**
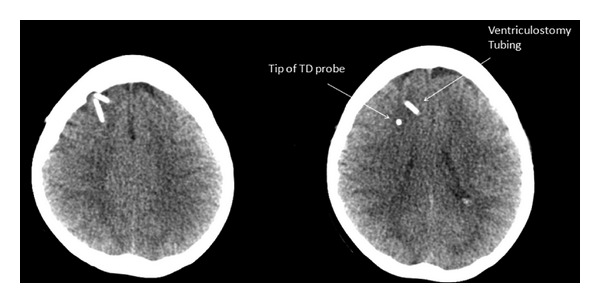
Postoperative head CT scan demonstrating the position of the thermal diffusion blood flow probe.

**Figure 2:**
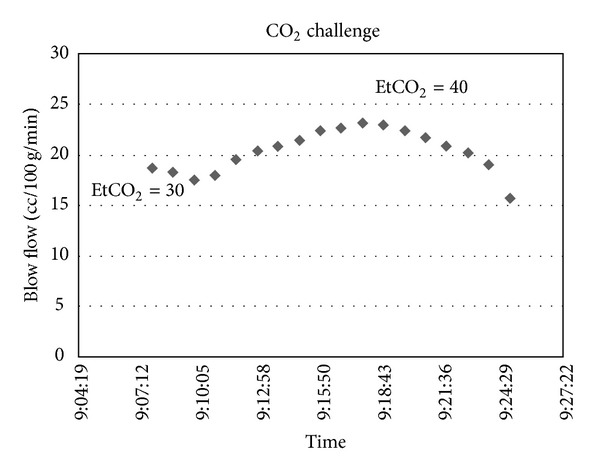
A typical tracing of thermal diffusion CBF measurement in response to change in end-tidal CO_2_.

The study protocol was approved by the University of Iowa Institutional Review Boards. In this prospective study, we used two complementary methods to investigate the effects of head position on CBF in SAH patients. SAH patients who underwent placement of ventriculostomy and thermal diffusion CBF monitor were included prospectively. Thermal diffusion probes (Hemedex, Cambridge, MA, USA) were inserted through the same burr hole as the ventriculostomy to a depth of 2 cm but at an angle so that the tip of the probe is away from the ventriculostomy tubing (Figure 1). Immediately after probe placement and before the patients were extubated, the end-tidal CO_2_ were adjusted within normal limit to check for associated change in CBF in order to verify proper functioning of the probe (Figure 2). Another group of SAH patients was also enrolled and underwent TCD studies only. Changes in head position and the corresponding changes in CBF parameters were evaluated. Specifically, on days 3, 7, and 10, the patient's CBF measurements (as measured by transcranial Doppler and thermal diffusion probe) were recorded in the supine and the 90-degree upright position 10 minutes later. TCD data were obtained in the medial cerebral arteries (MCA), the anterior cerebral arteries (ACA), and the posterior cerebral arteries (PCA) bilaterally using a handheld probe. Basic patient information such as age, sex, clinical exam, and hospital course was recorded. Delayed cerebral ischemia was defined as symptomatic vasospasm or infarction on CT attributable to vasospasm [11]. The percentage changes in mean blood flow velocity in each distribution from supine to sitting were calculated. Paired Student's *t*-test was used to determine statistical significance.

## 3. Results

The demographic details of the patients enrolled are shown in Table 1. Thirteen patients were enrolled, and the average age was 63 (ranging from 21 to 85). Seventy-seven percent (10/13) were females. Eight patients were studied with both thermal diffusion probe and TCD; five patients were studied with TCD only. The average Fisher grade was 3.3 ± 0.75 SD, and the average Hunt-Hess grade on admission was 2.5 ± 1.3 SD (ranging from 1 to 5). None of the patients had an adverse event with the manipulation of the head-of-bed. None of the patients developed delayed cerebral ischemia.

### 3.1. TCD Results

The MCA, ACA, and PCA were individually insonated bilaterally in both the supine and the upright sitting positions. The average increase in blood flow velocity from supine to sitting was 7.8% on day 3, 0.1% on day 7, and 13.1% on day 10. When each vessel was examined individually, the MCA appears to have the least changes in velocity depending on position (average 0.9% on day 3, −3.2% on day 7, and 1% on day 10). The ACA had 11.1% increase in velocity on day 3, −9.2% on day 7, and 24.2% on day 10. The PCA had 12.9% increase on day 3, 11.3% on day 7, and 14.7% on day 10. The absolute velocities were illustrated in Table 2. None of the velocity changes reaches statistical significance except for MCA changes on day 7 (*P* = 0.008). TCD value changes did not have any associated clinical manifestations, irrespective of Fisher or Hunt-Hess grade.

### 3.2. Thermal Diffusion CBF Measurement Results

#### 3.2.1. PCO_2_ Challenge and Regional CBF

Regional cerebral blood flow changed expectedly with changes in end-tidal PCO_2_ induced by adjusting ventilation (Figure 1). The regional cerebral blood flow increased with increased PCO_2_ and decreased with decreased PCO_2_. The average CBF changed from 13.7 to 23.6 cc/100 g/min with end-tidal PCO_2_ changes from 30 to 40 mmHg (*n* = 4).

#### 3.2.2. Postural Changes and Regional CBF

Thermal diffusion CBF measurement was done in 8 patients. The average CBF measurement in the supine position was 22.7 ± 10.3 mL/100 g/min. The average measurement in the 90-degree sitting position was 23.6 ± 9.1 mg/100 g/min. There was no statistically significant difference between the two groups (*P* = 0.196).

## 4. Discussion

Prolonged bed rest results in multiple physiological changes that could be detrimental. Supine positioning decreases tidal volume and minute ventilator volume [12, 13] and impairs the ability to clear secretions, resulting in atelectasis and pneumonia. Prolonged immobilization also results in negative nitrogen balance, calcium loss, diminished muscle strength, and orthostatic intolerance [10]. The risk of oxygen desaturation is higher in the supine position [14]. These changes are particularly pronounced in the elderly [15]. The rationale for supine positioning of SAH is to avoid hypoperfusion of the brain, especially considering the risk of delayed cerebral ischemia after SAH. However, several studies have shown that the incidence of clinical vasospasm is lower in the elderly [16, 17]. Therefore, it is unclear whether the risks of bed rest outweigh its presumed benefit, particularly in the older SAH population.

Zhang and Rabinstein [18] investigated the effects of HOB positioning on mean flow velocity in SAH patients using TCD. Measurements were taken for two HOB positions: first at 30°–45° and then at 0°– 15°. The authors found that HOB position did not significantly affect mean flow velocity and concluded that HOB position does not need to be specifically considered when interpreting the results of TCD studies in SAH patients. Blissitt et al. [19] also used TCD to study the effect of HOB elevation (at 20 and 45 degrees) on cerebrovascular dynamics in patients with mild or moderate vasospasm and found no consistent pattern of CBF changes. The measurements in both studies, however, were restricted to only one cerebral artery territory (MCA), the measurements were done in only one time point, and other modalities for cerebral perfusion assessment were not employed. The results of our study are in line with those of previous studies.

### 4.1. Implications

The current preference of restricting patients with SAH to only flat bed rest should be reconsidered, and HOB should be liberated pending changes in clinical exam. Changes in clinical examination when HOB is elevated are possibly suggestive of loss of autoregulation in these patients and/or early signs of vasospasm although this remains speculative and no such changes were observed herein.

### 4.2. Limitations

This study is limited by the small number of patients enrolled and that none of the patients developed delayed cerebral ischemia. The thermal diffusion probe provided continuous and absolute bedside measurement of regional CBF. However, the probe only samples a very small area in the white matter. We, therefore, performed TCD studies in multiple vascular territories as well in order to cross-validate the findings from these two complimentary methods.

## 5. Conclusion

We used two complimentary methods of CBF measurement to study cerebral hemodynamic in association with postural changes in the SAH patients. Changing HOB did not significantly affect either cerebral blood flow velocity or regional cerebral blood flow. These data suggest that early mobilization is not harmful and should be considered given the detrimental effects of prolonged bed rest.

## Figures and Tables

**Table 1:** Patients demographic.

Patient	Age	Sex	Fisher grade	WFNS	Hunt-Hess	Aneurysm location	Thermal diffusion probe
1	53	Female	2	1	1	pcom	y
2	72	Female	4	4	3	acom	y
3	74	Female	4	5	5	PICA	y
4	42	Female	3	1	2	pcom	y
5	63	Female	3	2	2	pcom	y
6	85	Female	4	2	3	acom	y
7	39	Female	4	1	2	acom	y
8	21	Female	2	1	1	pcom	y
9	77	Male	3	4	4	pcom	n
10	76	Male	3	2	3	acom	n
11	69	Male	4	2	4	acom	n
12	81	Female	4	2	2	acom	n
13	71	Female	3	1	1	acom	n

**Table:** (a)

Average velocity (cm/s)	Day 3		Day 7		Day 10	
	Supine	Upright	Supine	Upright	Supine	Upright
MCA	79	73	111	106	90	92
SD	56	41	49	46	46	52
ACA	69	72	72	62	56	68
SD	42	46	23	23	26	39
PCA	70	75	59	68	67	72

**Table:** (b)

Percentage changes from supine to upright (%)	Day 3	Day 7	Day 10
MCA	0.9	−3.2	1.0
ACA	11.1	−9.2	24.2
PCA	12.9	11.3	14.6
